# Daily intake of a dairy-based nutritional supplement improved self-reported gastrointestinal symptoms and modulated microbiota in adult Chinese volunteers

**DOI:** 10.1038/s41598-024-79360-9

**Published:** 2024-11-19

**Authors:** Klaudyna Borewicz, Yifan Zhao, Yaqiong Zhu

**Affiliations:** 1 Mead Johnson B.V., Middenkampweg 2, 6545 CJ Nijmegen, The Netherlands; 2 Mead Johnson Nutrition and Health Innovation Institute, 29/F, Yuexiu Financial Tower, No. 28 Zhujiang Road East, Zhujiang New Town, Guangzhou, 510623 People’s Republic of China; 3 Shenzhen Xbiome Biotech Co. Ltd, Shenzhen, 518055 People’s Republic of China

**Keywords:** Mild gastrointestinal symptoms, Constipation, Microbiome, High throughput sequencing, Healthy aging, Dietary supplement, Microbiology, Nutrition, Digestive signs and symptoms

## Abstract

Mild and persisting gastrointestinal symptoms (MPGS) as bloating, constipation or diarrhea are widespread in older adults and often accompanied by gut microbiota dysbiosis. Nutritional interventions help to regulate and restore microbiota and gut function. In this non-randomized continuous prospective cohort study, we evaluated the effects of a 21-day intervention using a dietary dairy-based supplement (AGIJOY™) on self-reported gastrointestinal (GI) symptoms, fecal microbiota composition and short chain fatty acids (SCFA) levels in a cohort of Chinese adults (N = 46, age 27–73) suffering from MPGS. Questionnaire data and fecal samples were collected at baseline (D0), on day 7 and 21 of intervention (D7 and D21). The results showed significant improvement in the self-reported GI symptoms on D21 and a gradual shift in the fecal microbiota composition. In the first week of intervention, the relative abundance (RA) of fecal bifidobacteria significantly increased and the RA of *Bacteroides* and *Helicobacter* decreased (*p* < 0.05). The levels of fecal SCFA remained unchanged during the study. To our knowledge, this is the first study testing the effect of this prebiotic and postbiotic containing milk-based supplement blend on gut microbiota and MPGS among Chinese population under a real living environment.

## Introduction

Mild and persisting gastrointestinal symptoms (MPGS) such as bloating, constipation or diarrhea are common manifestations of gastrointestinal (GI) disorders that include a range of functional gastrointestinal disorders (FGID; also referred as disorders of the gut-brain axis) and inflammatory bowel diseases (IBDs)^1^. The global prevalence of MPGS is high: a 2021 survey showed that over 40% of the 54,127 internet responders had FIGD symptoms, of which the prevalence of functional constipation affected almost 12% of participants, functional bloating /distention was reported by 3.5%, and diarrhea was reported by 4.7%. In China these rates were slightly lower and reached 34.4%, which included 10.6% subjects reporting constipation, 0.7% reporting functional bloating /distention and 5.6% reporting diarrhea^2^. Even though MPGS can affect people at any age, the percentage of people suffering from different forms of MPGS tends to increase with age^3^. As China has the largest ageing population in the world^4^, the high prevalence of MPGS impacts health, wellbeing, and performance of substantial proportion of citizens and have a considerable socioeconomic impact in terms of a premature senescence (i.e. earlier than expected onset of age-related physiological health decline) and medical burden. Besides physiological aging, other factors such as stress, alcohol use, smoking, medicine use, unhealthy diet, sedentary lifestyle, genetics, and co-existing diseases are important risk factors linked to GI disfunction, and they are also risk factors for gut microbiota dysbiosis^1–3,5–8^.

There are several ways for patients to manage MPGS and the most popular ways include over the counter medicine use (including herbal remedies), lifestyle changes such as exercise, meditation, and nutritional changes^8–11^. In more severe cases medical interventions are used to control MPGS^8,10^. Nutritional interventions can include diets rich in fiber or implementing elimination diets, and the use of dietary supplements. Since changes in microbiota are a hallmark of MPGS, many nutritional strategies aim to decrease dysbiosis and improve gut microbiota composition and function.

The milk-based supplement powder used in this study is available in Chinese markets and is designed to help control MPGS and it contains a prebiotic inulin and a yeast-based postbiotic (EpiCor®), as well as vitamins and minerals, lactoferrin, and easily digestible milk proteins. Inulin is a type of dietary fiber with a well-documented effect on microbiota and capable of improving bowel movement and stool consistency^12^, relieving constipation in elderly patients^13,14^, and enhancing gut barrier function during inflammatory conditions like IBD or Crohn’s disease^15,16^. The postbiotic has been shown to support a healthy gut function, modulate microbiome and immune system^17,18^. It also protects gut barrier function and gut mucosa structure^18,19^ and can induce anti-inflammatory effect^20,21^. As both these ingredients have the capability to modulate gut microbiota function, their health promoting effects are both local and systemic^21–35^.

Gastrointestinal health is essential for effective digestion and absorption of nutrients from diets, and any digestive dysfunction, especially chronic, has an impact on individuals’ nutritional status, energy levels and vitality. Nutritional deficiencies are especially common in the elderly, but their prevalence varies depending on socioeconomic status, nutritional habits, and geography^4^. In China, one of the most common deficiencies concerns protein intake. A 2022 study reported that regardless of age, the protein-energy malnutrition, as well as the vitamin A deficiency were the most common deficiencies, while protein-energy malnutrition and dietary iron deficiency had the highest age-standardized disability-adjusted life year (DALY) rates^36^. Iron deficiencies are also common and are associated with increased feeling of tiredness, mental fog, muscle weakness and, in severe cases, with anemia. Anemia is a serious problem in China and is associated with deficiency of iron and with low serum levels of vitamin B_12_. A recent study found that almost 29% of the surveyed adults 65 years or older in China had anemia^37^. Vitamin D deficiency can be a problem in northern regions of China and low levels of vitamin D are a risk factor for important medical and psychosocial problems, such as osteoporosis, sarcopenia, reduced immunity, or mood disorders including anxiety or depression^38^. Thus, we hypothesize that the macronutrients and micronutrients present in the supplement studied here could directly improve nutritional status and energy levels, as well as GI symptoms and microbiota composition and function in adults who regularly consume the supplement. As such, the nutritional interventions can offer an attainable approach to manage and improve general well-being, regulate digestion, and to lessen mild GI symptoms in older adults and in general, to contribute to a better nutritional status of the population.

Here we used a non-randomized continuous prospective cohort study to evaluate the effects of a 21-day intervention on subjective GI symptoms including fecal frequency and consistency, bloating and energy levels in a cohort of 46 Chinese adults (age 27–73) suffering from mild MPGS. We also evaluated the corresponding changes in the gut microbiota composition and short chain fatty acids (SCFA).

## Results

Of the 194 individuals who applied for the study, 81 were excluded based on the inclusion and exclusion criteria. Of the remaining 113 qualified, 55 subjects started the intervention on D0. Of those 54 completed 7 days intervention and 51 completed 21 days intervention. Subjective survey data was collected by 46 participants. Fecal samples from 5 individuals were not analyzed due to the missing data points on either D7 or D21. Ten subjects were excluded from the analyses of microbiota and SCFA due to having type 2 diabetes (T2D), hyperlipidemia, or they reported medicine or microbial ecological agents (e.g. probiotics) use within the two weeks prior to the enrollment. The final participant demographic data is summarized in Table 1.

**Table 1:** Characteristics of participants who completed the subjective surveys (N = 46) and the subgroup of fecal samples for the microbiota and SCFA analyses (n = 31).

	Total participants (N = 46)		Fecal sample donors (n = 31)	
	Frequency	Percentage (%)	Frequency	Percentage (%)
Sex				
Male	8	17%	8	26%
Female	38	83%	23	74%
Age				
20–30 years	6	13%	6	19%
31–40 years	10	22%	9	29%
41–50 years	12	26%	8	26%
51–60 years	14	30%	7	23%
61–70 years	3	7%	1	3%
71–80 years	1	2%	0	0%

### Clinical symptoms

No adverse effects were reported in this study, except a slight and transient increase in flatulence reported by 21% of participants at some point during the study. The self-reported clinical symptoms significantly changed over the 21-day study period, except for Bristol scores (Supplementary Table S2). The first significant improvement reported already on D7 of intervention was the feeling of being more energized, while the effect on fecal frequency and bloating were observed after D21. Five participants reported no changes (positive or negative) after intervention for any of the clinical parameters. Three participants reported increased bloating (reduced bloating score) or decreased energy with no other changes or improvements in the remaining clinical parameters. All other participants observed at least one clinical improvement during the intervention period, with most participants reporting at least two clinical benefits. The most reported improvement over the 21 days compared to baseline was the increased energy (50% participants), reduced bloating (41.3% of participants), improved stool consistency (21.74% of participants) and increased fecal frequency (19.57% of participants) (Table 2).

**Table 2:** Percent of subjects experiencing improvement, decline or no change in clinical symptoms during intervention periods: D0 to D7, D7 to D21 and D0 to D21.

N = 46	D0-D7				D7-D21				D0-D21			
Clinical effect	Freq.(%)	Gas (%)	Energy (%)	Bristol (%)	Freq. (%)	Gas (%)	Energy (%)	Bristol (%)	Freq. (%)	Gas (%)	Energy (%)	Bristol (%)
Improvement	15.22	21.74	36.96	26.09	6.52	36.96	23.91	23.91	19.57	41.30	50.00	21.74
Worsening	6.52	21.74	8.70	17.39	2.17	8.70	10.87	17.39	4.35	13.04	17.39	8.70
No effect	78.26	56.52	54.35	56.52	91.30	54.35	65.22	58.70	76.09	45.65	32.61	69.57

**Fig. 1:**
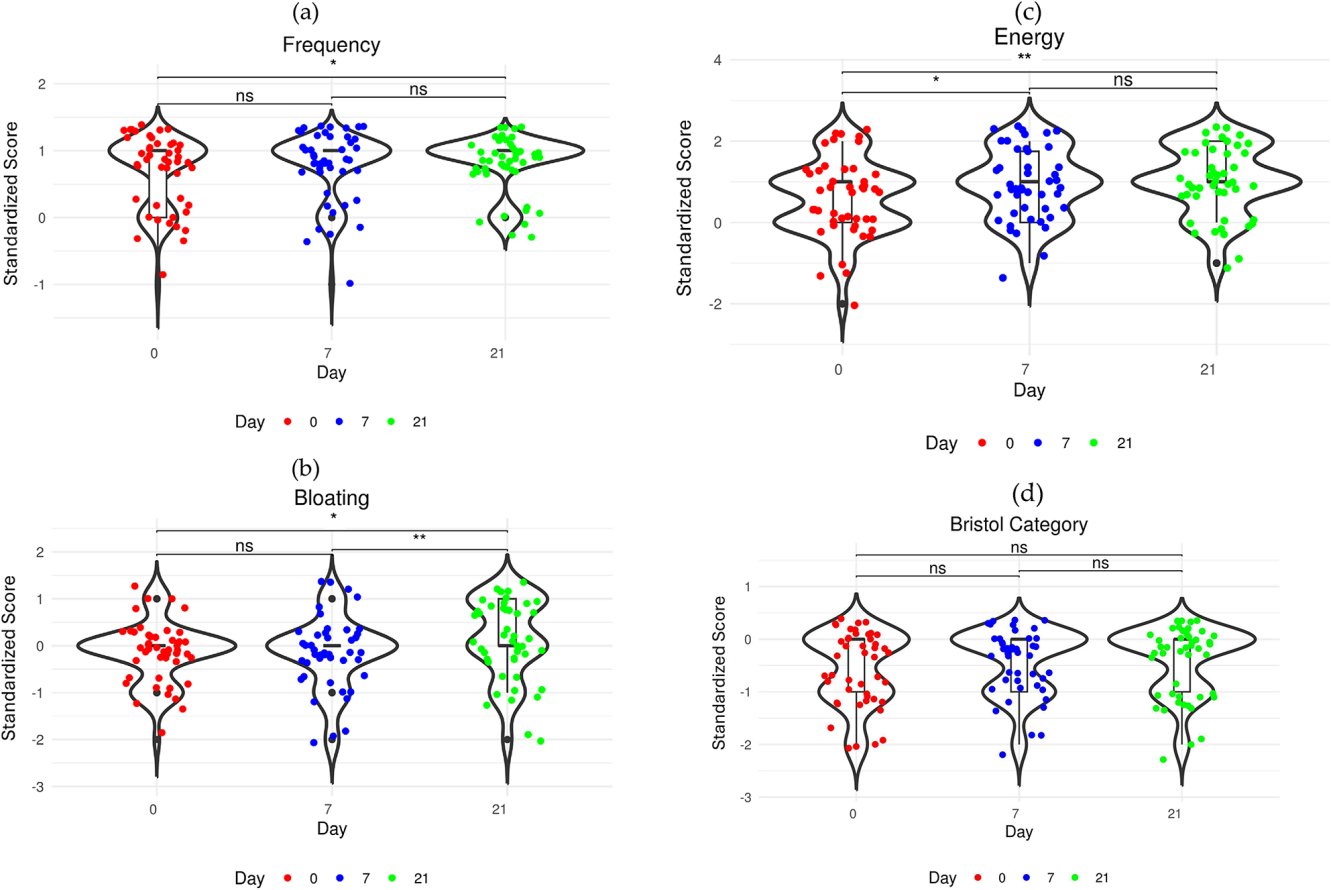
Violin plots with box plots of standardized scores at D0, D7 and D21; (**a**) Frequency; (**b**) Bloating; (**c**) Energy; (**d**) Bristol category score. Statistical significance was calculated using Wilcoxon signed-rank test with *p* values corresponding to ∗  ∗  < 0.01, ∗  < 0.05, ns ≥ 0.05.

The group average fecal frequency score increased from 0.67 at baseline (D0: 1, 0.67 ± 0.52), through 0.76 (D7: 1, 0.76 ± 0.48) and to 0.83 at the end of the study (D21: 1, 0.83 ± 0.38). This suggests that, on average, most participants experienced more regular daily stools, as compared to less frequent stools at the study baseline. A significant difference was observed after 21 days (Fig. 1a). Bloating remained stable in the first 7 days (D0: 0, − 0.15 ± 0.63, and D7: 0, − 0.17 ± 0.74) indicating mild to moderate bloating and then bloating significantly decreased between D7-21 (D21: 0, 0.13 ± 0.86) indicating mild to no bloating was observed (Fig. 1b). Participants also reported significant increase in energy levels after D7 and D21 of intervention as compared to the baseline, with a progressive improvement over time (D0: 1, 0.58 ± 0.91; D7: 1, 0.91 ± 0.84; D21: 1, 1.02 ± 0.83; Fig. 1c). Fecal consistency score was standardized with the zero-score indicating normal consistency and values below zero indicating abnormal stools, either watery or hard. During the study the average scores increased compared to baseline (D0: − 1, − 0.59 ± 0.69; D7: 0, − 0.46 ± 0.62; D21: 0, − 0.41 ± 0.62), suggesting improvement in the stool consistency (Fig. 1d).

### High throughput sequencing

**Fig. 2:**
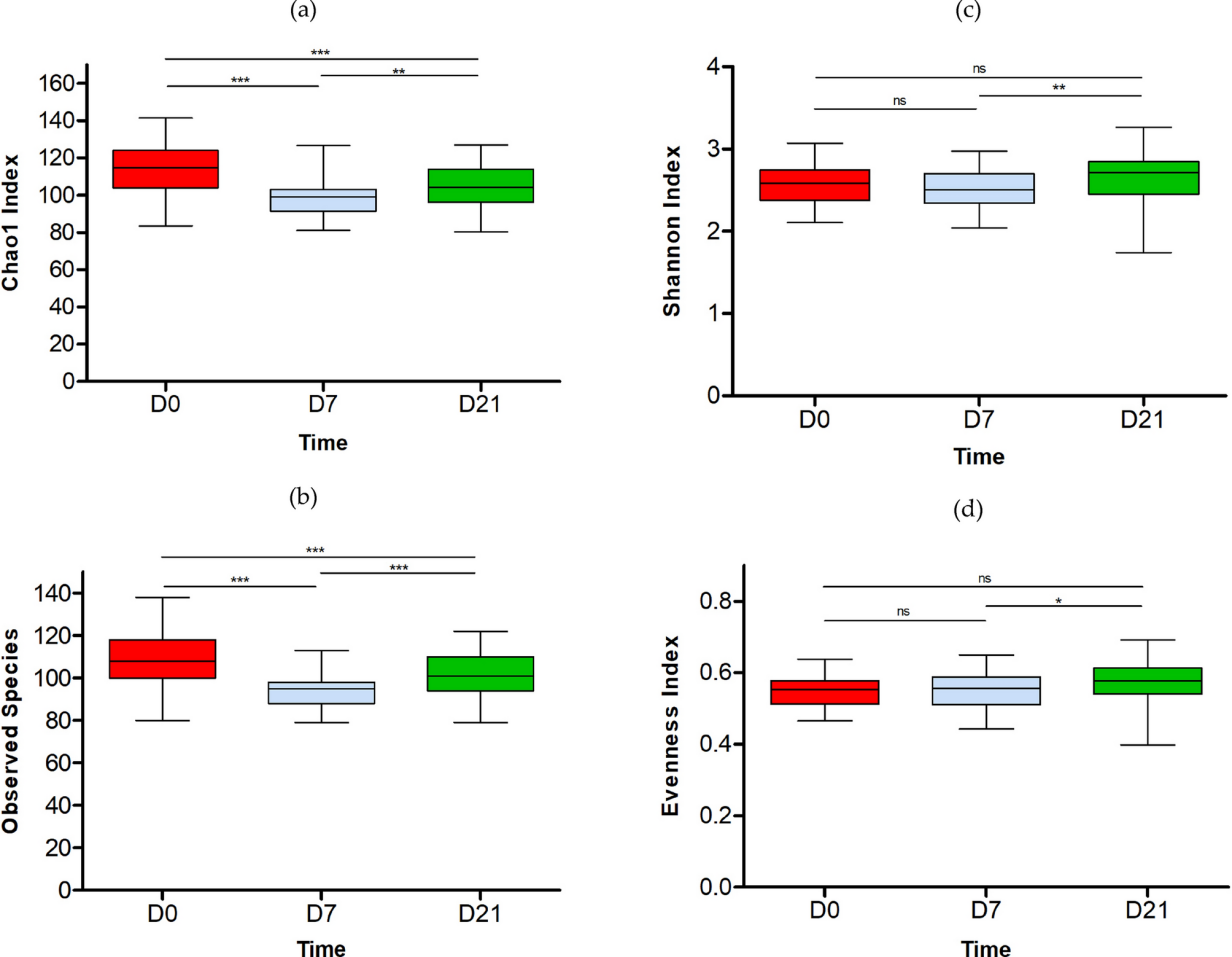
Alpha diversity box plots at D0, D7 and D21 (n = 31). Values reported are median, mean ± SD; (**a**) Chao1 (D0: 114.9, 115.5 ± 13.4; D7: 99.0, 99.0 ± 11.1; D21: 104.2, 105.0 ± 12.1); (**b**) Observed species ((genera); D0: 108.0, 109.3 ± 13.57; D7: 95.0, 94.2 ± 8.2; D21: 101.0, 100.4 ± 10.3); (**c**) Shannon diversity (D0: 2.59, 2.59 ± 0.26; D7: 2.50, 2.49 ± 0.23; D21: 2.71, 2.64 ± 0.29); (**d**) Evenness (D0: 0.55, 0.55 ± 0.05; D7: 0.56, 0.55 ± 0.05: D21: 0.58, 0.57 ± 0.06). Statistical significance calculated using Wilcoxon signed-rank test with p values corresponding to ∗  ∗  ∗  < 0.001, ∗  ∗  < 0.01, ∗  < 0.05, ns ≥ 0.05.

A total of 93 fecal samples were analyzed from participants for whom all three timepoints (baseline day 0 before intervention, day 7 and day 21 after the intervention) were available (n = 31). A total of 316 genus level taxa were identified (D0: 257 total detected genera; D7:216 total detected genera; D21: 241 total detected genera), the most abundant genera across all timepoints were *Bacteroides, Bifidobacterium, Faecalibacterium, Blautia, Prevotella, Lachnospiraceae unclassified*. Alpha diversity calculations were carried out on the genus level data and the resulting indices were compared across timepoints using Wilcoxon signed-rank test. Both Chao1 and Observed species differed significantly between timepoints and on D21 showed decrease in richness and diversity of taxa during intervention (Fig. 2a,b), while there was a significant increase in Shannon diversity and Evenness index between D7 and D21 (Fig. 2c,d). The PERMANOVA analysis of Bray–Curtis distances (abundance weighted measure) showed that microbiota composition was significantly different between timepoints (D0vs.D7: FDRp = 0.010, D7vs.D21: FDRp = 0.048, D0vs.D7vs.D21: FDRp = 0.024), however between D0vs.D21 there was no significant difference (FDRp = 0.302). PCoA plot of beta diversity of the gut microbiota based on Bray–Curtis distances and with samples color coded by time point is presented in the Supplementary Figure S1.

**Fig. 3:**
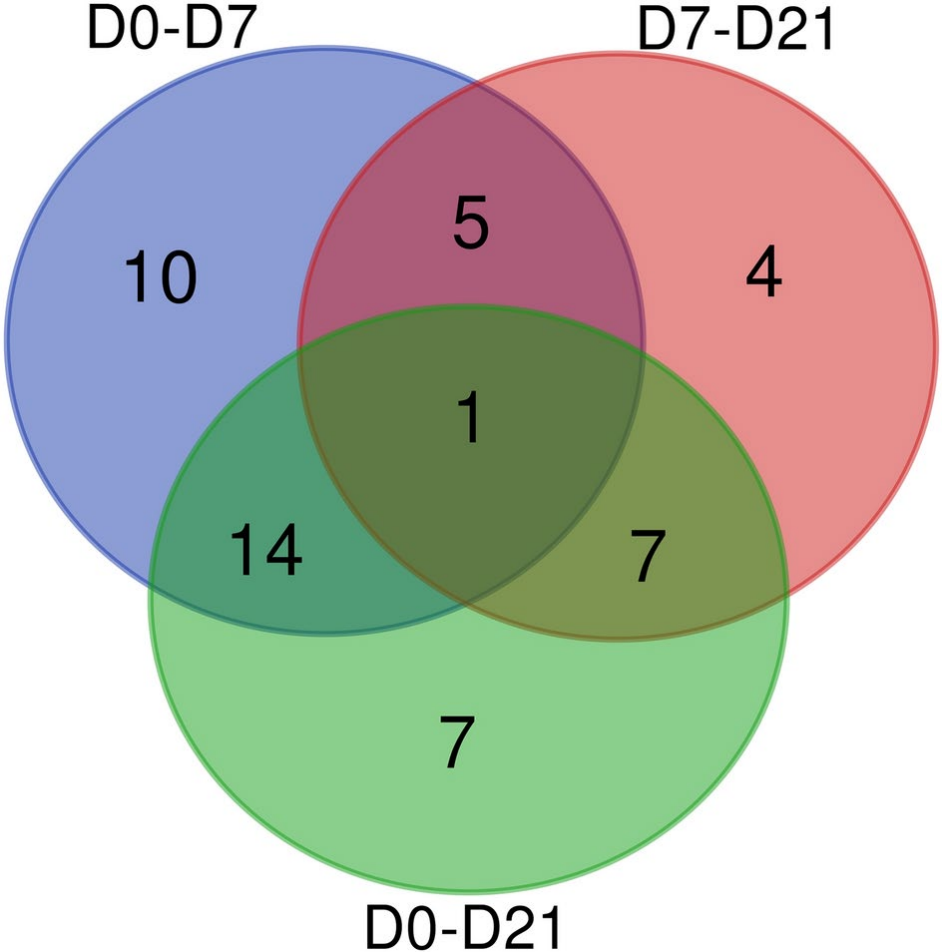
Venn diagram showing the numbers of differentially abundant genera between different timepoints, as identified in Kruskal Wallis analysis (FDRp < 0.05).

Average RA at each sampling point and differentially abundant taxa identified in the Kruskal–Wallis analyses were reported in Supplementary Table S1. Compared to baseline levels (D0), after D7 and D21 there was a significant (FDRp < 0.05) change in RA of 30 and 29 genera respectively. However, only 15 of the same genera stayed significantly different from baseline through both D7 and D21, while the remaining genera were only different from the baseline at either D7 (15 genera) or at D21 (14 genera). Between D7 and D21 there was a significant change in RA of 17 genera. Overall, different genera exhibited different relative abundance patterns in responses throughout the study, such as steady increase or decrease in RA, increase followed by decrease or decrease followed by increase in RA. Figure 3 shows a Venn diagram of the numbers of differentially abundant genera between different timepoints, and Supplementary Table S1 shows their average RA and the FDR corrected and uncorrected *p*-values.

**Fig. 4:**
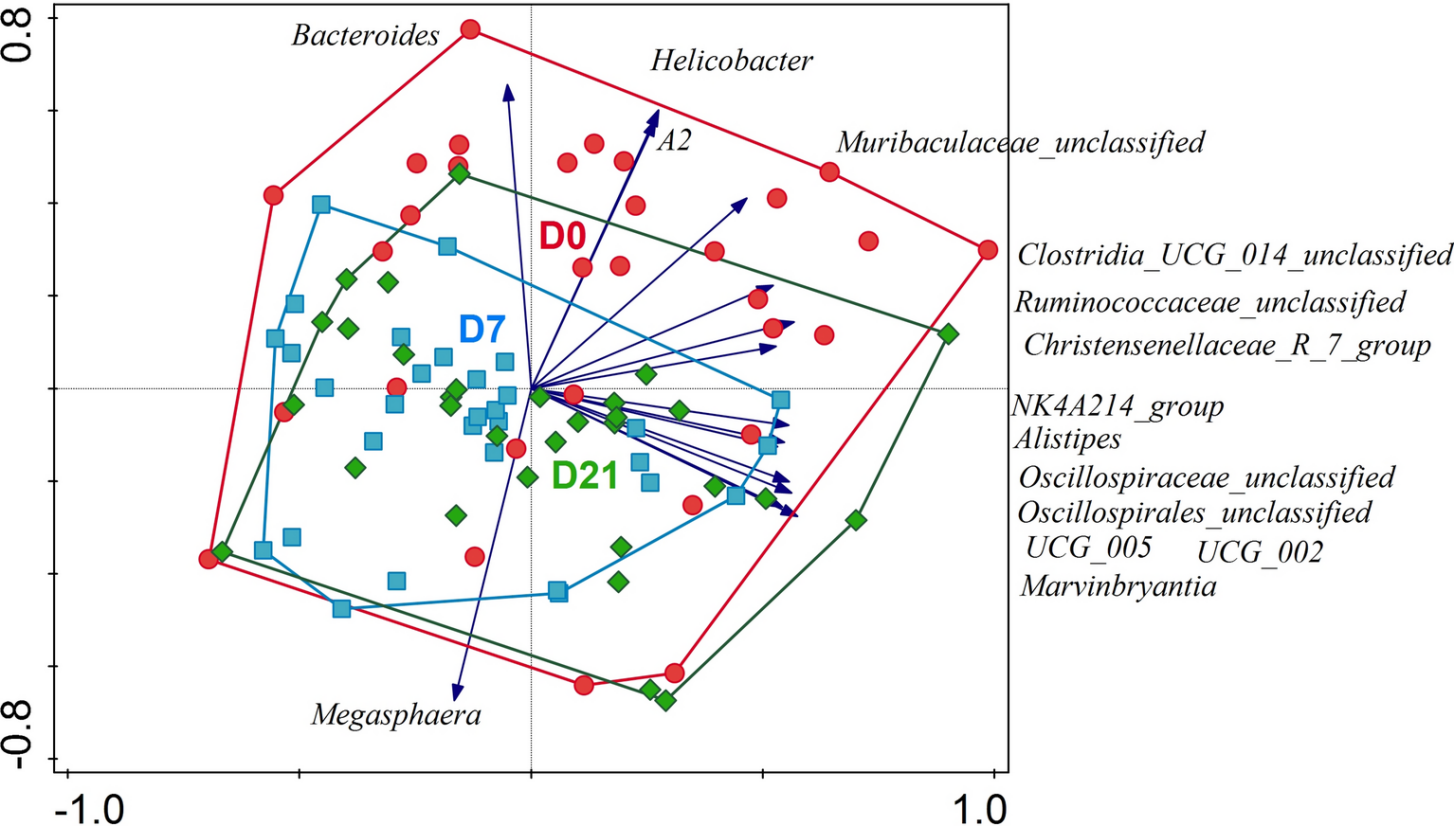
PCA analysis of log transformed genus level relative abundance data. Samples colored and enveloped by timepoint (red—D0, blue—D7, green—D21) showing directional shift in relation to experimental timepoint. Ten best fitting taxa are displayed, and their increasing abundance is indicated with blue vectors.

After the first seven days of supplementation there was a significant (*p* < 0.05) increase in the RA of the core genera, such as *Bifidobacterium* from 13.5% (D0) to 21.7% (D7) followed by a slight decrease to 17.4% (D21). This change was accompanied by a significant decrease in genus *Bacteroides* from 20.3% (D0), to 14.0% (D7) and 17.5% (D21). The RA of *Faecalibacterium*, the third most abundant genus in the dataset, remained stable across timepoints with the RA of about 12%. *Blautia*, the fourth most abundant group, increased significantly between D7 and D21 from 7.0% to 9.0%. During the first seven days and over the course of the study there was a significant decrease in *Helicobacter*, that was no longer detected on D7 and D21. Genus level relative abundance data was used in the multivariate PCA and RDA analyses. The PCA analysis showed no clear groupings of samples but the color coding of samples by sampling point revealed a slight directional shift associated with sampling time and a decline in RA of *Bacteroides* and an increase in RA of *Megasphaera* (Fig. 4).

**Fig. 5:**
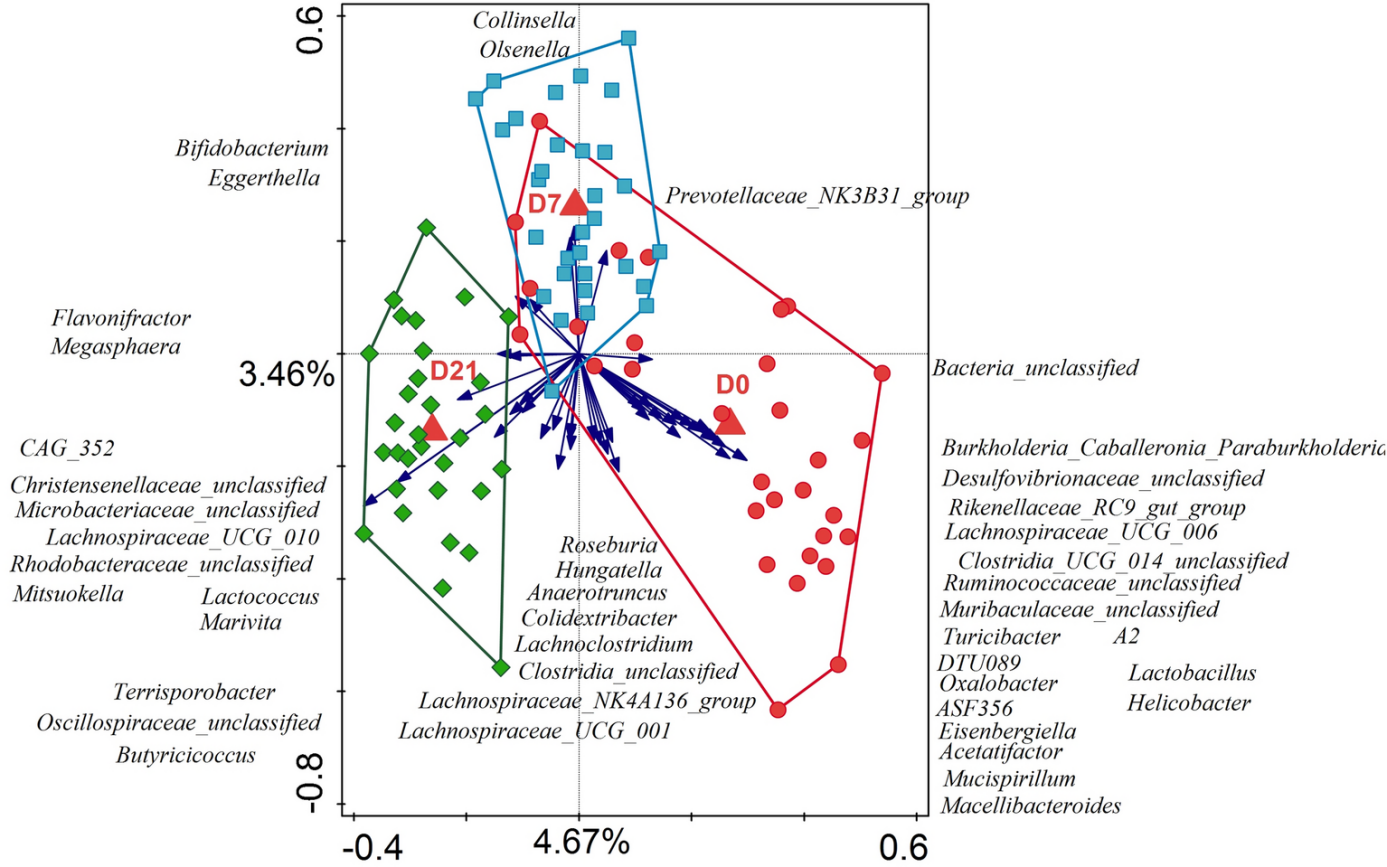
Constrained partial RDA analysis with covariates, using the log transformed genus level relative abundance data at each time point. Samples are color coded and enveloped by timepoint (red—D0, blue—D7, green—D21) and 45 best fitting genus level taxa are displayed with their increasing abundance indicated by the direction of the blue vectors. Group centroids are indicated with red triangles.

Partial RDA analyses on combined data across all timepoints and using participant’s gender or age as explanatory variables revealed significant association (FDR *p* = 0.001) of gender and age with microbiota composition, thus these variables were included as confounders in subsequent analyses. Bloating and subjective energy level were significantly associated with microbiota composition (FDR *p* = 0.001 and FDR *p* = 0.024, respectively). There was no significant association detected between microbiota and the remaining clinical measures: stool frequency (FDR *p* = 0.233), or Bristol stool category (FDR *p* = 0.265). Partial RDA analysis with timepoints as explanatory variable, and gender and age as confounders, showed a clear clustering of samples at each timepoint (Fig. 5). The relative abundance of genera was significantly associated with the duration of intervention (FDR *p* = 0.001), and it explained 4.9% variation in microbiota on D0, 4.8% on D7 and 3.9% on D21. The top 45 best fitting genera contributing to sample separation in this RDA analysis are displayed on the RDA plot (Fig. 5), and the majority of these genera were the same as the differentially abundant genera identified by the Kruskal–Wallis analyses (Supplementary Table S1).

### Short chain fatty acids

The analyses of SCFA showed that acetate was the dominating SCFA across timepoints, followed by propionate and butyrate. Total stool SCFA levels varied between participants and ranged on D0: between 3,432.4 ng/mg of wet feces to 19,276.9 ng/mg, on D7: 5872.0 ng/mg to 22,006.3 ng/mg, and on D21: 2355.7 ng/mg to 20,426.0 ng/mg. At the end of the intervention 38.7% of participants had higher levels of total SCFA as compared to their baseline levels, and 61.3% had lower levels as compared to their baseline readouts. Butyrate levels remained stable, and per mg of wet feces they averaged on D0: 728.6 ng/mg (range: 130.5 to 1758.8 ng/mg), on D7: 698.4 ng/mg (range: 290.8 to 1216.6 ng/mg), and on D21: 729.9 ng/mg (range 327.5 to 2240.0 ng/mg). At the end of the study 43.3% of participants showed higher stool butyrate levels compared to their baseline levels and in 56.7% of the participants the butyrate levels decreased. The differences in total SCFA levels, concentrations of individual acids and thus, their relative amounts, did not pass the significance cutoff (*p* ≥ 0.05) (Table 3).

**Table 3:** The mean, median, standard deviation, standard error, and statistical differences in concentrations of different short chain fatty acids analyzed in fecal samples of participants (n = 31) at each sampling timepoint; D0, D7 and D21. ns ≥ 0.05.

		SCFA (ng/mg wet feces)				*p*-values		
	Time	Mean	Median	Std. Deviation	Std. Error	D0 vs. D7	D0 vs. D21	D7 vs. D21
Acetate	D0	8,987.6	9,446.0	3,497.5	628.2	ns	ns	ns
	D7	9,288.4	8,921.4	3,718.2	667.8	ns	ns	ns
	D21	8,705.9	8,614.8	3,541.6	636.1	ns	ns	ns
Butyrate	D0	728.63	687.9	342.4	61.5	ns	ns	ns
	D7	698.4	690.2	259.9	46.7	ns	ns	ns
	D21	729.9	657.3	406.9	73.1	ns	ns	ns
Caproate	D0	26.1	12.1	28.6	5.1	ns	ns	ns
	D7	21.2	11.9	33.3	6.0	ns	ns	ns
	D21	24.9	16.8	34.3	6.2	ns	ns	ns
Isovalerate	D0	503.7	406.8	451.6	81.1	ns	ns	ns
	D7	449.2	357.8	380.4	68.3	ns	ns	ns
	D21	523.7	456.3	493.9	88.7	ns	ns	ns
Isobutyrate	D0	301.3	283.6	142.5	25.6	ns	ns	ns
	D7	292.3	288.9	111.1	20.0	ns	ns	ns
	D21	306.3	274.4	176.5	31.7	ns	ns	ns
Propionate	D0	1,369.4	1,438.3	595.7	90.8	ns	ns	ns
	D7	1.384.1	1,447.8	353.1	63.4	ns	ns	ns
	D21	1,348.2	1,351.0	495.7	89.0	ns	ns	ns
Valerate	D0	200.4	146.7	206.5	37.1	ns	ns	ns
	D7	178.0	115.6	160.4	28.8	ns	ns	ns
	D21	243.9	161.6	311.7	56.0	ns	ns	ns
Total	D0	12,117.2	12,819.3	4,416.9	793.3	ns	ns	ns
	D7	12,311.6	11,741.0	4,035.5	724.8	ns	ns	ns
	D21	11,883.0	11,372.9	4,435.6	796.7	ns	ns	ns

### Biomarker analysis

Spearman correlation analysis was run to search for microbial and SCFA biomarkers of clinical symptoms. No significant (FDRp < 0.05) correlations were detected at individual sampling points, and for combined data across timepoints there was no significant correlation between SCFA concentrations and clinical scores, however the analysis showed that genus *Mitsuokella* was positively associated with bloating (FDRp = 0.00002, corr = 0.545). All SCFA showed statistically significant correlations with various genus level taxa, as summarized in Supplementary Figure S2.

## Discussion

The primary objective of this study was to investigate the effects of daily supplementation on the mild GI symptoms in otherwise healthy Chinese adults and elderly. In this study, we collected self-reported questionnaire data and fecal samples at baseline (D0), during (D7) and after (D21) the 21-day intervention. After the first week of intervention there were no significant changes in stool frequency, bloating and stool consistency, though 15.22% of participants reported increased stool frequency, 21.74% reported less bloating, and 26.09% reported better stool consistency (Table 2). Between D7 and 21 the stool frequency and consistency average scores were maintained at the same level (p ≥ 0.05), while bloating was significantly improved (*p* < 0.01; Fig. 1). Compared to the baseline, at the end of the study the statistically significant gastrointestinal improvements included reduced gassiness (41.3% of participants), improved stool consistency (21.74%) and fecal frequency (19.57%; Table 2). Fifty percent of the participants also reported feeling more energized at the end of the trial as compared to baseline. We did not collect any follow-up data in this study, thus, we cannot conclude if these beneficial effects persisted even after the supplementation use was discontinued.

The supplement’s modulatory effect on GI function can be associated with the presence of bioactive ingredients, especially the prebiotic and the postbiotic that have well-documented digestive health effect. Inulin is a type of fructan naturally found in many plants, of which chicory root, artichokes and garlic are the richest natural sources. Nowadays chicory root is used as the main commercial source of inulin^39^. Inulin has a very long history of research use, it is proven to be safe even at high doses, and its function as a dietary fiber has been known since the early nineties^40^. The effectiveness of inulin to relieve constipation in elderly has been first documented in a clinical trial already in 1997^14^ and inulin became one of the most studied prebiotics on the market. EpiCor® is made through anaerobic fermentation using baker’s yeast, *Saccharomyces cerevisiae.* The resulting fermentate contains numerous bioactive compounds, proteins, peptides, antioxidants, polyphenols, organic acids, nucleotides, polysaccharides (1–3 1–6, beta glucans), and mannans, which together have been shown to support a healthy gut function, modulate microbiome and immune system^17,18^. EpiCor® used at a dose of 500 mg/day has been shown to relieve constipation, improve fecal consistency and fecal frequency and mitigate symptoms such as bloating/distension or fullness in individuals suffering from reduced bowel movements and other symptoms of GI discomfort^41^.

Inulin and EpiCor® can modulate gut microbiota composition and function^20,41–44^. The microbiota prebiotic effect of inulin from chicory root has been already investigated since early 1950s^45^, even though the concept of “prebiotic” was only officially introduced in 1995^46^. Numerous in vitro and in-vivo studies confirmed that inulin can reshape gut microbiota composition, specifically increase counts and RA of stool bifidobacteria^14^, *Anaerostipes, Faecalibacterium*, and *Lactobacillus*^47^, decreases enterococci and enterobacteria^14^, and can decrease the RA of genus *Bacteroides*^47^. In our study we saw an increase in the average RA of bifidobacteria, and a corresponding decrease in the RA of Bacteroides, and in all participants the ratio of these two groups has changed through the study, meaning all participants could be classified as responders. We also saw a significant increase in the RA of *Anaerostipes* and a decrease in the RA of *Enterococcus*, but contrary to these earlier studies we did not detect significant changes in the RA of *Faecalibacterium* and *Escherichia-Shigella*, and we also noted a significant decrease in the RA of *Lactobacillus* (Supplementary Table 1). Inulin can increase production of short chain fatty acids (SCFA)^42^, especially butyrate production^14^ or overall production of short chain fatty acids (SCFA) and lower gut pH^39,42^. Microbiota and their metabolites influence the host via different “axis” and thus, fiber rich diets have been linked with healthy microbiota and maintaining physical and mental well-being during healthy aging^48,49^. The gut-brain axis can explain the link between the gut function, the microbiota, and mental well-being^50,51^. In most studies the function of prebiotics links to their effect on modulation of gut microbiota composition and microbial metabolites production^14,19,20,39,41–45,47,48,50–60^. In our study we observed a clear gradual and directional shift in the fecal microbiota composition of the participants’ stools (Figs. 4 and 5) and a corresponding change in alpha diversity (Fig. 2). However, we did not detect any changes in the fecal SCFA levels.

In the first week of intervention, we saw a significant decrease in the number (richness) of observed genus level taxa followed by a small yet statistically significant increase on D7-21. However, over the whole study period there was a significant decrease in the number of detected genera (Fig. 2b), with the average number decreasing by 8.5 genera during the study period of D0-21. The evenness index and Shannon index value increased significantly between D7 and D21, which was in agreement with an earlier study where inulin supplementation in elderly participants resulted in long term significant increase in alpha diversity, as measured with Shannon index^59^. We also observed significant changes in the relative abundance of genus level taxa, including both the core and the minor groups (Fig. 5). Based on the PERMANOVA and Kruskal Wallis analyses we saw a significant change in RA, presence (or absence) of species occurring from D0 to D7 and a further change from D7 to D21, however the D21 microbiota seemed, at least partially, to resemble the pre- intervention state, as the significance between D0vs.D21 was lost (FDRp = 0.302). Further analyses showed that the response speed and direction of change varied across genera, with some groups responding early, late or throughout the study, and some genera responding with only increase, only decrease or undergoing significant fluctuations in abundance between timepoints (Fig. 3, Supplementary Table S1). A recent study showed that inulin can be bound and degraded by a wide range of gut bacteria, and the resulting fermentation byproducts participate in trophic chains, thus the community-wide effect and time dependent changes in the population would be expected^58^.

The most abundant taxa in this study cohort at baseline were *Bacteroides* (D0 average RA 20.3%), *Bifidobacterium* (D0 average RA 13.5%), *Faecalibacterium* (D0 average RA 12.5%), *Blautia* (D0 average RA 8.1%), and *Prevotella* (D0 average RA 6.6%, Supplementary Table S1). The distribution was different than that reported in another recent study that surveyed fecal microbiota in Chinese adults and showed that fecal microbiota in that study cohort was dominated by *Bacteroides* (average RA 30.38%), *Prevotella* (average RA 11.72%) and *Faecalibacterium* (average RA 9.72%) while the average RA of *Bifidobacterium* and *Blautia* reached approximately only 2% and 3%, respectively. The study also showed that fecal composition differed between individuals and was influenced by enterotype, diet and other lifestyle factors^61^. In our study we did not collect any data on lifestyle and dietary intake, thus we cannot explain the differences in microbiota, nor we can account for impact of these factors in our data analyses. Nevertheless, it is interesting to note, that our study population had already a very high starting bifidobacteria level that was approaching the upper known levels of 15% for adults^57^. After the first week of intervention (D7), we observed even further significant increase in *Bifidobacterium* (average RA 21.7%) and a significant decrease in *Bacteroides* (average RA 13.9%), and between D7 and D21 also a significant increase in *Blautia* (average RA 9.0%). High levels of bifidobacteria and *Blautia* have been associated with healthy aging and longevity^49,62^. The levels of *Prevotella* and *Faecalibacterium* did not change (Supplementary Table S1). These results were expected, and similar findings had been reported in earlier in vitro and in vivo studies on the microbiota modulation and bifidogenic effect of either inulin^14,20,47,59,63^ or EpiCor®^20,43^. Furthermore, it has been shown that consuming inulin in the morning rather than evening might have stronger effects on microbiota in older adults^56^ and in our study participants were advised to consume the product in the mornings. Also, as baseline microbiota composition could influence response to prebiotic, in particular the *Bacteroides/Bifidobacterium* ratio^55^, thus we can speculate that the relatively high baseline levels of bifidobacteria could contribute to very fast and significant increase in RA of *Bifidobacterium* on D7. It is interesting to note that none of these high abundance/high prevalence genera mentioned above were identified as biomarkers of any of the clinical symptoms measured in this study. Instead, a low abundance, glucose and other carbohydrate fermenting genus, *Mitsuokella,* was identified to correlate positively (corr = 0.47, FDRp = 0.000059) with bloating scores. *Mitsuokella* also significantly increased in RA between D0 to D21 (from 0.00027 to 0.00285; FDRp = 0.005), and D7 to D21 (from 0.00041 to 0.00285; FDRp = 0.013; Supplementary Table S1) which is also the time when bloating scores increased significantly (Fig. 1b). This genus has been shown to produce SCFA (propionate, ammonia, butyrate) and gas in-vitro^64^.

One of the best studied mechanisms via which gut microbiota benefits the host is by the production of various SCFA. Butyrate and other SCFA play important roles in regulating gut barrier function and peristaltic activity of the intestines^65^. In our study the average concentrations of individual SCFA and the total SCFA in wet feces remained constant throughout the study (*p* > 0.05; Table 3). On D21 41% of participants had numerically higher levels of total SCFA as compared to their baseline levels, and 59% had lower levels as compared to their baseline readouts. Butyrate levels also remained stable, and at the end of the study 53% of participants showed numerically higher stool butyrate levels compared to their levels from D0, and in 47% of participants butyrate levels on D21 were numerically lower than at the baseline. Our results do not agree with previous findings indicating that fermentation of either inulin or EpiCor® tends to produce butyric acid in vitro^20,63^ and in vivo^54^, though for inulin this effect has not always been consistent^59^. In our study the high individual variability and small number of participants could have been a reason we did not detect any statistically significant differences in any of the SCFA. In addition, it is known that the fecal SCFA amounts do not represent the actual SCFA amounts produced through microbial fermentation in the gut, but instead they are only the fraction of SCFA which has not been absorbed or otherwise utilized by the host and its microbiome. It is estimated that only 5–10% of SCFA is excreted in feces^53^, thus the actual levels produced could have been much higher but went undetected in our trial. These reasons could also possibly explain why we could not detect statistically significant association between SCFA concentrations and clinical symptoms when running Spearman correlation analysis during our search for biomarkers.

Already at the first week of the intervention there was a significant increase in the self-reported average energy levels, with nearly 37% of participants reporting more energy on D7, and 50% at the end of the study compared to baseline. There are different factors which indirectly or directly could contribute to energy levels. Normalizing GI function and improving gut microbiota composition and activity are important factors in facilitating digestive processes and thus, increasing the uptake of nutrients. The supplement tested here can also provide direct nutritional support. A 25 g sachet contains 5.8 g milk protein, as well as 11.5 g carbohydrates, 3.4 g fats, vitamins, and minerals. AGIJOY™ also contains an amino acid taurine, which has been shown to increase muscle performance, cardiac function, liver activity, and metabolism of adipose tissue, however the ability of the body to make taurine is limited. Thus, taurine deficiencies have been linked with dysfunction in energy metabolism^66^. Most Chinese adults and elderly do not meet the minimum recommended nutrient intake (RNI) for protein and on average consume less protein than the recommended (65 g/day for male adults aged above 18 years and 55 g/day for female adults aged above 18 years (1.0 g/kg/day))^67^. Adequate protein intake is important for the energy balance, body mass maintenance, muscle strength and physical performance, but also to improve well-being and lowering the risk of age-related diseases via controlling oxidation and lowering inflammation and their downstream catabolic effects^67^. AGIJOY™ also contains vitamins C, B_12_, D, iron and magnesium that are essential for metabolic processes in the body, as they impact cell functions and metabolism and their adequate levels can prevent feeling of tiredness, mental and physical fatigue, low energy, muscle weakness, mood changes, or even more serious health conditions, such as anemia. Thus, a regular supplementation of macro and micronutrients could have a profound effect on energy and health status of older adults.

This study had some limitations. The main limitations were the low number of participants and that the subjects were recruited only from Guangdong area, thus the population used in this study might not be fully representative of all groups inhabiting other regions of China that vary in their dietary and lifestyle habits. Another limitation was that we did not include blood collections to analyze serum levels of the SCFA and other blood parameters. We followed subjects for only 21 days thus we could not measure any long-term health effects of using this supplement. Subjects’ age was also a confounding factor influencing gut microbiota and clinical status. We expect some results could vary in different age subgroups, however due to the small sample size we were unable to detect any pre-post intervention differences in gut microbiota composition and SCFAs in age stratified subgroups. Therefore, studies on a larger number of participants and including participants from different regions of the country and across various age groups might be beneficial, especially when considering the detection of effects on gut microbiota structure and function. Another limitation of this study is the lack of the control group; therefore, this pre-post intervention study design might be considered inferior when compared to the randomized controlled trials. However, our study represents a close to a real-world circumstance where people who are not clinically diagnosed with GI disease but still frequently experience mild GI problems reach out for over-the-counter nutritional support to remedy their GI issues. This study showed that despite limitations, we were still able to observe a significant improvement in gut symptoms in such a short-term intervention period, also considering a lower content of prebiotic inulin and postbiotic EpiCor® than previous studies. We expect that these results could be even more pronounced if a randomized controlled study design was implemented, with a long-term intervention and a post-intervention follow-up.

## Materials and methods

### Subjects recruitment and study design

Study participants were recruited from Guangdong, China through social media platforms and screened based on the inclusion and exclusion criteria as previously reported^41^. The primary objective was to include volunteers who were over the age 18, able and willing to follow the study protocol, male or female, and self-reporting presence of GI symptoms in the one month prior the study, but without clinically diagnosed GI diseases. The symptoms ranged from mild, moderate to severe and included either reduced bowel movements with an average 2–5 stools/week, or bloating ranging from mild flatulence (score with slight discomfort) to severe flatulence (strong pain and restricted activity). The exclusion criteria included the history of severe GI/hepatic, hematological/immunologic, metabolic disorders, endocrine disorders, celiac disease or type I diabetes mellitus, high fever at the time of enrolment, or currently undergoing or planning to undergo a surgery (including major abdominal surgery interfering with GI function), laboratory assessments, or dialyses. Participants with type 2 diabetes or hyperlipidemia, or those taking medicines or microbial ecological agents (e.g. probiotics) in the 2 week period prior to the enrollment were excluded from the microbiota and SCFA analyses.

Eligible participants were offered consent forms and the 21-day supply of the formulated milk powder AGIJOY™, AJcore® fecal collection kits (Catalog No. NA-012-T, AJcore, CN) and were instructed about the method of collecting and mailing fecal samples. Subjects were instructed to have one package (25 g) of the supplement daily in the morning with breakfast for three weeks. Daily sachet contained 20 g bovine milk powder, 1.5 g inulin, 250 mg EpiCor®, 12.5 mg lactoferrin, and vitamins and minerals. The nutrients in the formula are listed in Table 4.

**Table 4:** Nutritional composition in daily serving of the milk-based supplement formula.

Nutrients	Value/package(25g)
Energy	431 kJ
Protein	5.8 g
Fat	3.4 g
Carbohydrate	11.5 g
Fiber (inulin)	1.5 g
Sodium	75 mg
Vitamin A	135 mcg RE
Vitamin D	2.1 mcg
Vitamin E	3.5 mg α-TE
Vitamin B_2_	0.2 mg
Vitamin B_12_	0.3 mcg
Vitamin C	19.0 mg
Folic acid	100 mcg DFE
Phosphorus	125 mg
Potassium	200 mg
Magnesium	12.5 mg
Calcium	300 mg
Iron	2.0 mg
Zinc	1.50 mg
Selenium	5.0 mcg
Manganese	0.06 mg
Taurine	7.5 mg
L-carnitine	7.5 mg
Lactoferrin	12.5 mg
EpiCor®	250 mg

Questionnaire data and fecal samples were collected at baseline (D0), after 7th day intervention (D7) and after 21st day intervention (D21). During the intervention, the participants were not allowed to take any medicines that would impact the result, to take supplements containing prebiotics, probiotics or postbiotics, or to change dietary habit and lifestyle. Daily intake of the formula was recorded in the form of social media (WeChat group) to reduce the rate of loss-to-follow-up. Adverse events were also recorded ^68^. Fecal samples were collected using the AJcore® fecal collection kit (Catalog No. NA-012-T, AJcore, CN) delivered by subjects in ice box and stored at -80 °C until further analysis.

### Genomic DNA extraction and bacterial 16S rRNA sequencing and microbiota analysis

DNA was extracted from fecal samples according to the protocol provided in the QIAamp PowerFecal DNA Kit (Cat. No. 51804, Qiagen). The V4 region of the 16S rRNA gene was amplified using primers (515F: 5'-GTGCCAGCMGCCGCGGTAA -3' and 806R: 5'-GGACTACNNGGGTATCTAAT-3', Invitrogen, Carlsbad, CA, USA) with 12 bp barcode. PCR reactions, containing 25 µL2x Premix Taq (Takara Biotechnology, Dalian Co. Ltd., China), 1 µL each primer(10 μM) and 3 µL DNA (20 ng/ µL) template in a volume of 50 µL, were amplified by thermocycling: 5 min at 94 °C for initialization; 30 cycles of 30 s denaturation at 94 °C, 30 s annealing at 52 °C, and 30 s extension at 72 °C; followed by 10 min final elongation at 72 °C (BioRad S1000, Bio-Rad Laboratory, CA, USA). The length and concentration of the PCR product were detected by 1% agarose gel electrophoresis. Libraries were prepared from targeted PCR products mixed in equal amounts according to the GeneTools Analysis Software (Version4.03.05.0, SynGene; https://www.bioinformatics.com.cn/srplot)^68^ and further purified with E.Z.N.A. Gel Extraction Kit (Omega, USA). Sequencing libraries were generated using NEBNext® UltraTM II DNA Library Prep Kit for Illumina® (New England Biolabs, MA, USA) following manufacturer’s recommendations and index codes were added. The library quality was assessed on the Qubit® 2.0 Fluorometer (Thermo Fisher Scientific, MA, USA) and the final library was sequenced on an Illumina Nova6000 platform for 250 bp paired-end reads.

Raw reads were demultiplexed and the primers were removed with Cutadapt v3.4 (https://cutadapt.readthedocs.io/en/v3.4/)^69^. DADA2 version 1.22 (https://benjjneb.github.io/dada2/index.html)^70^ was applied downstream for quality filtering (Q > 30), reads merging, chimera removal and ASV generation. Samples were rarefied at 30,000 reads per sample. Each unique ASV was assigned to a high- resolution taxonomy using the Ribosomal Database Project classifiers (implemented in DADA2) and SILVA Database v138 (https://www.arb-silva.de/documentation/release-138/)^71^. Sequencing data were deposited at SRA with project number PRJNA1085899. The resulting contaminant-free 16S rRNA reads were used for calculating relative abundance and the following statistical analyses. The genus-level relative abundance was calculated by summing up the relative abundances of ASVs belonging to each genus on the phylogenic tree.

### Short-chain fatty acids (SCFA) measurement

A total of seven SCFA were measured, including acetic acid, propionic acid, butyric acid, valeric acid, isobutyric acid, isovaleric acid, and caproic acid for the 31 fecal sample donors at D0, D7 and D21. A volume of 400 µL of extraction solution (methanol: acetonitrile = 2:1, v:v) was added to approximate 50 mg fecal samples in addition to magnetic beads for homogenization and grinding (50 Hz for 120 s). Magnetic beads were removed by centrifuging at 4°C, 20,000g for 3 min. A volume of 60 µL pre-cooled precipitation reagent (methanol: acetonitrile = 2:1, v:v) was added to 20 µL supernatant. The mixture was vortexed for 5 min, incubated at -20°C for 4 h, followed by centrifuging at 4°C, 20,000g for 15 min. Forty microliters of the supernatant were taken and 20 µL of 3-NPH (3-Nitrophenylhydrazine) and 20 µL of EDC (N-(3-Dimethylaminopropyl)-N′-ethylcarbodiimide) were added. The mixture was vortexed for 1 min, incubated for 30 min followed by the addition of 80 µL of 10% acetonitrile. A volume of 90 µL was taken from the mixture and added with 90 µL of ultrapure water. After mixing and filtering, the filtrate was centrifuged at 4°C, 3000g for 5 min. The resulting supernatant was used for the SCFA measurement using Waters UPLC system (Waters, USA) coupled to a QTRAP6500 (SCIEX, Canada; https://sciex.com/products/software/multiquant-software)^72^ equipped with an ESI source and operated in the negative-ion mode. Chromatographic separations were performed on a Waters BEH C18 (2.1 × 50 mm, 1.7 mm) UPLC column, in which solvent A (water: formic acid; 100:0.01, v/v) and solvent B (acetonitrile: formic acid; 100:0.01, v/v) were used as the mobile phase for gradient elution. The column flow rate was 0.35 mL/min. All quantification data was processed using the MultiQuant 2.0 software (SCIEX, Canada, https://sciex.com/products/software/multiquant-software)^72^.

### Statistical analyses

Self-reported clinical parameters included fecal frequency, bloating in the last week, energy status in the last week, and stool consistency as described in the Bristol Stool Form^73^. The questionnaire scores were standardized for further analyses, with higher score indicating more favorable conditions (Table 5). Median, mean, and standard deviation and standard errors of each clinical score at each timepoint was calculated and reported and the Wilcoxon signed-rank test was run in R using function ggpubr (v.0.4.0; https://www.rdocumentation.org/packages/ggpubr/versions/0.4.0/topics/stat_compare_means) to compare scores between different time points, with a p < 0.05 considered significant (Supplementary Table S2). Violin plots were used to plot scores for each clinical measure reported by each participant at each timepoint ^74^.

**Table 5:** Score assignment for questionnaire data.

	Standardized score
Fecal frequency in past week	
1/week	− 1
2–3/week	0
4–5/week	0
6–7/week	1
More than 8 times	1
Bloating in past week	
0: no bloating	1
1: mild bloating	0
2: moderate bloating with distinct feeling and a little discomfort	− 1
3: stronger than moderate bloating with distinct feeling and a little pain	− 2
4: severe with extreme bloating, along with pain and restricted activity	− 3
Feeling energized in past week	
0: tired	− 2
1: somewhat tired	− 1
2: normal	0
3: somewhat energized	1
4: energized	2
Bristol stool score in the past week	
Type 1	− 2
Type 2	− 2
Type 3	− 1
Type 4	0
Type 5	0
Type 6	− 1
Type 7	− 2

Fecal microbiota alpha and beta diversities were computed utilizing the R (v. 3.6.3; https://cran-archive.r-project.org/bin/windows/base/old/3.6.3/) package Phyloseq (v. 1.30.0; https://joey711.github.io/phyloseq/)^75^. Alpha diversity calculations were carried out on the genus level data. The Chao1 index and Observed genus were used to represent the richness, and the Shannon index was used to present both the richness and evenness of each sample. The Wilcoxon signed-rank test was run in R using function ggpubr to compare the alpha diversity between different timepoints with significance cutoff of p < 0.05. A permutational multivariate analysis of variance (PERMANOVA) was performed using the adonis function in Vegan package (https://github.com/vegandevs/vegan)^76^ in R, on Bray–Curtis dissimilarity distance to compare microbiota composition at consecutive sampling points. Permutation number was set at 999 and FDR corrected *p*-values were calculated with a significance cutoff set at FDR *p* < 0.05.

Genus level relative abundance (RA) data was used to identify differentially abundant genera between timepoints using Kruskal–Wallis test using QIIME (v. 1.9.1; http://qiime.org/home_static/dataFiles.html)^77^. Unconstrained (PCA) and constrained (RDA) multivariate analyses were carried out in Canoco5 (v. 5.15; http://canoco5.com/index.php)^78^. To reduce the noise in the data, prior to analyses 205 genera that were low abundance (highest RA reported in any sample equal or less than 0.0001) and /or low prevalence (less than 5% prevalence in the total set) were grouped into “other” category and included alongside the high abundance high prevalence genera (n = 166). Prior to the Kruskal–Wallis, RDA and PCA analyses the final RA genus data was log transformed. In the resulting PCA and RDA plots, each point indicates one sample and the proximity of points in the two-dimensional space corresponds to similarity in sample microbial composition. The taxa vectors point towards the samples in which the RA of specified taxa was the highest, while the vector lengths correspond to R- squared calculated by dividing the taxa scores by their SD^78^. To allow better visibility only the top ten best fitting taxa were displayed in PCA plot, and in RDA plots the top forty-five best fitting taxa were displayed as those also comprised most of the differentially abundant taxa identified in the Kruskal–Wallis analyses (FDR *p* < 0.05). The best fitting taxa were those that explained the highest percentage of variation in the relative abundance within the ordination axes. In the RDA analyses, the explanatory variables tested were time of sample collection (D0, D7, D21), participant age and gender, bloating score, energy score, standardized Bristol score and fecal frequency score. The significance of the explanatory variables was assessed using Monte Carlo permutation test at 499 random permutations^78^. For each SCFA and for total SCFA concentrations statistical significance between timepoints was calculated using Wilcoxon signed-rank test. Spearman correlations were calculated in R stats (R 3.6.3; https://www.r-project.org/) to identify correlations between genus level taxa and SCFA and between microbial and SCFA biomarkers for each clinical outcome, with the significance cutoff for the correlations set at FDRp < 0.05.

## Supplementary Information

Supplementary Information.

## Data Availability

Raw sequencing data was deposited at SRA with project number PRJNA1085899.
