# Supplementary material for: Daily intake of a dairy-based nutritional supplement improved self-reported gastrointestinal symptoms and modulated microbiota in adult Chinese volunteers
Source: Sci Rep. 2024 Nov 19;14:28651. doi: 10.1038/s41598-024-79360-9 (PMC11576911; doi:10.1038/s41598-024-79360-9)
Supplement: Supplementary file 1 — Supplementary Information. [file 41598_2024_79360_MOESM1_ESM.docx]

**Supplementary Tables**

Supplementary Table S1. Average relative abundance of genus level taxa at each sampling point. Differentially abundant taxa between timepoints were identified using Kruskal-Wallis test and corresponding p-values and FDR corrected p-values are shown. Dots indicate lack of significance (p -values and FDR p-values > 0.05).

|  | **AVG _ RA (n=41)** | | | **D0 vs D7 vs D21** | | **D0 vs D7** | | **D7 vs 21** | | **D0 vs 21** | |
| --- | --- | --- | --- | --- | --- | --- | --- | --- | --- | --- | --- |
| **Genus** | **D0** | **D7** | **D21** | **p-values** | **FDRp** | **p-values** | **FDRp** | **p-values** | **FDRp** | **p-values** | **FDRp** |
| *Bacteroides* | 0.20325 | 0.13983 | 0.17547 | . | . | 0.02565 | . | . | . | . | . |
| *Bifidobacterium* | 0.13535 | 0.21658 | 0.17378 | 0.05480 | . | 0.01907 | . | . | . | . | . |
| *Faecalibacterium* | 0.12540 | 0.12277 | 0.12478 | . | . | . | . | . | . | . | . |
| *Blautia* | 0.08152 | 0.06993 | 0.09027 | . | . | . | . | 0.02018 | . | . | . |
| *Prevotella* | 0.06636 | 0.08558 | 0.06129 | . | . | . | . | . | . | . | . |
| *Lachnospiraceae_unclassified* | 0.03499 | 0.02927 | 0.03864 | . | . | . | . | 0.02256 | . | . | . |
| *Agathobacter* | 0.02748 | 0.02344 | 0.02732 | . | . | . | . | . | . | . | . |
| *Fusicatenibacter* | 0.02022 | 0.01982 | 0.02620 | . | . | . | . | . | . | . | . |
| *Ruminococcus* | 0.02009 | 0.01713 | 0.01601 | . | . | . | . | . | . | . | . |
| *Anaerostipes* | 0.01882 | 0.02103 | 0.02728 | 0.04662 | . | . | . | . | . | 0.02832 | . |
| *Megamonas* | 0.01776 | 0.01344 | 0.01092 | . | . | . | . | . | . | . | . |
| *Collinsella* | 0.01610 | 0.04587 | 0.00971 | 0.00025 | 0.00166 | 0.00066 | 0.00538 | 0.00029 | 0.00459 | . | . |
| *Lachnospira* | 0.01601 | 0.01422 | 0.01347 | . | . | . | . | . | . | . | . |
| *Erysipelotrichaceae_UCG_003* | 0.01503 | 0.01109 | 0.01097 | . | . | . | . | . | . | . | . |
| *Subdoligranulum* | 0.01199 | 0.01524 | 0.01500 | . | . | . | . | . | . | . | . |
| *Fusobacterium* | 0.01021 | 0.00158 | 0.00168 | 0.01516 | 0.05353 | . | . | 0.03416 | . | 0.00719 | 0.04423 |
| *Dorea* | 0.00935 | 0.00837 | 0.00945 | . | . | . | . | . | . | . | . |
| *Alistipes* | 0.00904 | 0.00455 | 0.00705 | . | . | . | . | . | . | . | . |
| *Coprococcus* | 0.00865 | 0.00717 | 0.00897 | . | . | . | . | . | . | . | . |
| *Roseburia* | 0.00855 | 0.00545 | 0.00701 | 0.00159 | 0.00854 | 0.00064 | 0.00538 | 0.01149 | . | . | . |
| *Lachnoclostridium* | 0.00790 | 0.00608 | 0.01037 | 0.02760 | . | 0.02340 | . | 0.01943 | . | . | . |
| *Sutterella* | 0.00750 | 0.00455 | 0.00472 | . | . | . | . | . | . | . | . |
| *Phascolarctobacterium* | 0.00633 | 0.01047 | 0.00688 | . | . | . | . | . | . | . | . |
| *Romboutsia* | 0.00611 | 0.00734 | 0.00378 | . | . | . | . | . | . | . | . |
| *Parasutterella* | 0.00557 | 0.00259 | 0.00263 | . | . | . | . | . | . | . | . |
| *Clostridia_UCG_014_unclassified* | 0.00527 | 0.00108 | 0.00147 | 0.00424 | 0.02012 | 0.00123 | 0.00874 | . | . | 0.01863 | . |
| *UCG_002* | 0.00493 | 0.00385 | 0.00384 | . | . | . | . | . | . | . | . |
| *Parabacteroides* | 0.00465 | 0.00431 | 0.00630 | . | . | . | . | . | . | . | . |
| *Lachnospiraceae_NK4A136_group* | 0.00457 | 0.00320 | 0.00552 | 0.00556 | 0.02563 | 0.00182 | 0.01241 | 0.03407 | . | . | . |
| *Alloprevotella* | 0.00390 | 0.00325 | 0.00349 | 0.00020 | 0.00148 | 0.00090 | 0.00669 | . | . | 0.00033 | 0.00285 |
| *Megasphaera* | 0.00390 | 0.00258 | 0.00499 | 0.00033 | 0.00209 | 0.01190 | . | 0.00949 | . | 0.00092 | 0.00693 |
| *Lachnospiraceae_ND3007_group* | 0.00357 | 0.00432 | 0.00608 | 0.05090 | . | . | . | 0.05373 | . | 0.01597 | . |
| *Escherichia.Shigella* | 0.00354 | 0.00355 | 0.00583 | . | . | . | . | . | . | . | . |
| *Catenibacterium* | 0.00354 | 0.00363 | 0.00076 | 0.00001 | 0.00011 | . | . | 0.00010 | 0.00195 | 0.00001 | 0.00015 |
| *Streptococcus* | 0.00335 | 0.00878 | 0.00586 | . | . | . | . | . | . | . | . |
| *Lactobacillus* | 0.00319 | 0.00126 | 0.00072 | 3.40E-06 | 0.00003 | 0.00002 | 0.00031 | . | . | 0.00002 | 0.00020 |
| *Lachnospiraceae_UCG_004* | 0.00282 | 0.00244 | 0.00306 | . | . | . | . | . | . | . | . |
| *Oscillospirales_unclassified* | 0.00244 | 0.00203 | 0.00285 | . | . | . | . | . | . | . | . |
| *Enterococcus* | 0.00236 | 0.00061 | 0.00101 | 0.00158 | 0.00854 | 0.02002 | . | . | . | 0.00026 | 0.00242 |
| *Ruminococcaceae_unclassified* | 0.00228 | 0.00063 | 0.00052 | 0.00293 | 0.01430 | 0.00075 | 0.00582 | . | . | 0.01823 | . |
| *Monoglobus* | 0.00228 | 0.00183 | 0.00224 | . | . | . | . | . | . | . | . |
| *Akkermansia* | 0.00222 | 0.00188 | 0.00231 | . | . | . | . | . | . | 0.05000 | . |
| *Muribaculaceae_unclassified* | 0.00218 | 0.00050 | 0.00219 | 0.00587 | 0.02634 | 0.00543 | 0.03300 | . | . | 0.01178 | . |
| *Butyricicoccus* | 0.00217 | 0.00195 | 0.00262 | 0.00037 | 0.00226 | 0.01604 | . | 0.00012 | 0.00220 | . | . |
| *CAG_56* | 0.00209 | 0.00222 | 0.00287 | . | . | . | . | . | . | . | . |
| *Butyricimonas* | 0.00198 | 0.00083 | 0.00151 | . | . | . | . | . | . | . | . |
| *UCG_005* | 0.00186 | 0.00092 | 0.00131 | . | . | . | . | . | . | . | . |
| *Tyzzerella* | 0.00185 | 0.00089 | 0.00140 | . | . | . | . | . | . | . | . |
| *Burkholderia_Caballeronia_*  *Paraburkholderia* | 0.00177 | 0 | 0 | 3.89E-12 | 1.30E-10 | 5.37E-08 | 2.97E-06 | . | . | 8.23E-08 | 2.76E-06 |
| Other n=205 | 0.00173 | 0.00094 | 0.00114 | . | . | . | . | . | . | . | . |
| *Christensenellaceae_R_7_group* | 0.00172 | 0.00093 | 0.00234 | 0.04399 | . | . | . | 0.01169 | . | . | . |
| *Prevotellaceae_NK3B31_group* | 0.00170 | 0.00650 | 0.00081 | 3.92E-07 | 4.65E-06 | 0.00016 | 0.00206 | 1.33E-06 | 0.00005 | 0.00667 | 0.04256 |
| *Holdemanella* | 0.00141 | 0.00287 | 0.00181 | 0.00181 | 0.00938 | . | . | 0.00062 | 0.00900 | 0.01824 | . |
| *Lachnospiraceae_UCG_001* | 0.00140 | 0.00077 | 0.00161 | 0.01067 | 0.04217 | 0.00724 | 0.04097 | 0.01786 | . | . | . |
| *Barnesiella* | 0.00140 | 0.00052 | 0.00092 | . |  | . | . | . | . | . | . |
| *Clostridium_sensu_stricto_1* | 0.00137 | 0.00167 | 0.00190 | . | . | . | . | . | . | . | . |
| *Paraprevotella* | 0.00137 | 0.00118 | 0.00112 | . | . | . | . | . | . | . | . |
| *Intestinibacter* | 0.00136 | 0.00043 | 0.00102 | . | . | . | . | . | . | . | . |
| *Klebsiella* | 0.00114 | 0.00463 | 0.00228 | 0.02943 | . | 0.03004 | . | . | . | 0.02432 | . |
| *Lachnospiraceae_FCS020_group* | 0.00113 | 0.00110 | 0.00133 | . | . | . | . | . | . | . | . |
| *Dialister* | 0.00113 | 0.00100 | 0.00175 | 0.01472 | 0.05311 | 0.02983 | . | 0.00514 | 0.04804 | . | . |
| *Incertae_Sedis* | 0.00112 | 0.00122 | 0.00191 | . | . | . | . | . | . | . | . |
| *Bilophila* | 0.00105 | 0.00127 | 0.00151 | . | . | . | . | . | . | . | . |
| *NK4A214_group* | 0.00099 | 0.00048 | 0.00122 | . | . | . | . | . | . | . | . |
| *Oscillospiraceae_unclassified* | 0.00093 | 0.00057 | 0.00106 | 0.03492 | . | . | . | 0.01056 | . | . | . |
| *Erysipelatoclostridium* | 0.00087 | 0.00054 | 0.00087 | . | . | . | . | . | . | . | . |
| *Lachnospiraceae_UCG_010* | 0.00080 | 0.00066 | 0.00126 | 0.00903 | 0.03747 | . | . | 0.00303 | 0.03012 | 0.02725 | . |
| *Marvinbryantia* | 0.00080 | 0.00057 | 0.00058 | . | . | . | . | . | . | . | . |
| *Haemophilus* | 0.00074 | 0.00113 | 0.00059 | . | . | . | . | . | . | . | . |
| *UCG_003* | 0.00073 | 0.00054 | 0.00093 | . | . | . | . | . | . | . | . |
| *Syntrophococcus* | 0.00053 | 0.00031 | 0.00054 | . | . | . | . | . | . | . | . |
| *Turicibacter* | 0.00049 | 0.00018 | 0.00024 | 9.86E-07 | 0.00001 | 2.62E-06 | 0.00005 | . | . | 0.00003 | 0.00033 |
| *Desulfovibrio* | 0.00049 | 0.00021 | 0.00061 | . | . | . | . | . | . | . | . |
| *Flavonifractor* | 0.00046 | 0.00059 | 0.00103 | 0.01052 | 0.04217 | . | . | . | . | 0.00255 | 0.01840 |
| *Colidextribacter* | 0.00045 | 0.00027 | 0.00050 | 0.01975 | . | 0.04059 | . | 0.00899 | . | . | . |
| *Terrisporobacter* | 0.00045 | 0.00002 | 0.00045 | 0.00004 | 0.00027 | 0.00058 | 0.00538 | 0.00001 | 0.00030 | . | . |
| *Actinomyces* | 0.00042 | 0.00015 | 0.00009 | . | . | . | . | . | . | . | . |
| *CAG_352* | 0.00041 | 0.00032 | 0.00068 | 4.93E-08 | 6.29E-07 | . | . | 1.21E-06 | 0.00005 | 1.41E-06 | 0.00003 |
| *Helicobacter* | 0.00039 | 0 | 0 | 3.92E-12 | 1.30E-10 | 5.43E-08 | 2.97E-06 | . | . | 8.30E-08 | 2.76E-06 |
| *Oscillibacter* | 0.00038 | 0.00032 | 0.00044 | . | . |  | . | . | . | . | . |
| *RF39_unclassified* | 0.00037 | 0.00014 | 0.00015 | . | . | 0.03768 | . | . | . | . | . |
| *Odoribacter* | 0.00037 | 0.00020 | 0.00035 | . | . |  | . | . | . | . | . |
| *Hungatella* | 0.00037 | 0.00002 | 0.00019 | 0.00002 | 0.00017 | 4.01E-06 | 0.00007 | 0.00088 | 0.01097 | . | . |
| *A2* | 0.00034 | 0 | 0 | 3.89E-12 | 1.30E-10 | 5.37E-08 | 2.97E-06 | . | . | 8.27E-08 | 2.76E-06 |
| *Christensenellaceae_unclassified* | 0.00034 | 0.00023 | 0.00095 | 2.10E-06 | 0.00002 | . | . | 5.57E-06 | 0.00018 | 0.00005 | 0.00045 |
| *Prevotellaceae_UCG_001* | 0.00034 | 0.00186 | 0.00221 | 0.00622 | 0.02716 | . | . | 0.01647 | . | 0.00440 | 0.02923 |
| *Methanobrevibacter* | 0.00033 | 0.00056 | 2.15E-06 | . | . | . | . | . | . | . | . |
| *Mitsuokella* | 0.00027 | 0.00041 | 0.00285 | 0.00157 | 0.00854 | . | . | 0.00124 | 0.01319 | 0.00869 | 0.04977 |
| *Senegalimassilia* | 0.00027 | 0.00022 | 0.00017 | . | . | . | . | . | . | . | . |
| *Erysipelotrichaceae_unclassified* | 0.00024 | 0.00011 | 0.00016 | . | . | . | . | . | . | 0.02942 | . |
| *Coprobacter* | 0.00021 | 0.00007 | 0.00010 | . | . | . | . | . | . | . | . |
| *Family_XIII_AD3011_group* | 0.00020 | 0.00015 | 0.00022 | . | . | . | . | . | . | . | . |
| *Eggerthella* | 0.00019 | 0.00050 | 0.00041 | 0.01151 | 0.04344 | 0.00528 | 0.03300 | . | . | 0.01818 | . |
| *Clostridia_unclassified* | 0.00019 | 0.00009 | 0.00020 | 0.00134 | 0.00793 | 0.00320 | 0.02101 | 0.00090 | 0.01097 | . | . |
| *Lachnospiraceae_AC2044_group* | 0.00018 | 0.00016 | 0.00013 | . | . | . | . | . | . | . | . |
| *UCG_010_unclassified* | 0.00018 | 0.00011 | 0.00029 | . | . | . | . | . | . | . | . |
| *Acidaminococcus* | 0.00017 | 0.00035 | 0.00035 | . | . | . | . | . | . | . | . |
| *UBA1819* | 0.00017 | 0.00017 | 0.00020 | . | . | . | . | . | . | . | . |
| *Negativibacillus* | 0.00017 | 0.00009 | 0.00016 | . | . | . | . | . | . | . | . |
| *Desulfovibrionaceae_unclassified* | 0.00017 | 1.08E-06 | 9.68E-06 | 1.29E-10 | 3.56E-09 | 1.31E-07 | 5.37E-06 | . | . | 6.61E-07 | 0.00002 |
| *Coriobacteriales_Incertae_Sedis_*  *unclassified* | 0.00015 | 0.00015 | 0.00014 | . | . | . | . | . | . | . | . |
| *Adlercreutzia* | 0.00015 | 0.00018 | 0.00012 | . | . | . | . | 0.03989 | . | . | . |
| *Lachnospiraceae_UCG_006* | 0.00013 | 9.68E-06 | 6.45E-06 | 1.51E-09 | 2.79E-08 | 1.32E-06 | 0.00004 | . | . | 1.59E-06 | 0.00003 |
| *ASF356* | 0.00013 | 0 | 0 | 3.58E-10 | 8.49E-09 | 9.52E-07 | 0.00003 | . | . | 5.89E-07 | 0.00002 |
| *Victivallis* | 0.00013 | 0.00002 | 0.00020 | . | . | . | . | 0.01710 | . | . | . |
| *Faecalitalea* | 0.00011 | 0.00010 | 0.00008 | . | . | . | . | . | . | . | . |
| *Veillonella* | 0.00011 | 0.00020 | 0.00009 | . | . | . | . | 0.03420 | . | . | . |
| *GCA_900066575* | 0.00010 | 0.00008 | 0.00010 | . | . | . | . | . | . | . | . |
| *Intestinimonas* | 0.00010 | 0.00003 | 0.00010 | . | . | . | . | . |  | . | . |
| *Prevotellaceae_unclassified* | 0.00010 | 0.00043 | 0.00020 | 0.03633 | . | . | . | . | . | 0.00827 | 0.04901 |
| *Eisenbergiella* | 0.00009 | 0.00001 | 0.00002 | 0.00004 | 0.00027 | 0.00019 | 0.00220 | . | . | 0.00092 | 0.00693 |
| *Olsenella* | 0.00009 | 0.00024 | 0.00008 | 0.00024 | 0.00163 | 0.00034 | 0.00368 | 0.00115 | 0.01301 | . | . |
| *Tuzzerella* | 0.00009 | 0.00006 | 0.00013 | . | . | . | . | . | . | . | . |
| *Clostridia_vadinBB60_group_*  *unclassified* | 0.00009 | 0.00002 | 0.00007 | 0.04473 | . | 0.01417 | . | . | . | . | . |
| *Shuttleworthia* | 0.00009 | 0.00004 | 0.00013 | . | . |  |  | . | . | . | . |
| *Anaerovoracaceae_unclassified* | 0.00009 | 0.00005 | 0.00008 | . | . |  |  | . | . | . | . |
| *Family_XIII_UCG_001* | 0.00008 | 0.00002 | 0.00007 | 0.03052 | . | 0.00842 | 0.04605 | . | . | . | . |
| *Bacteroidales_unclassified* | 0.00008 | 0.00006 | 0.00006 | . | . | . | . | . | . | 0.03106 | . |
| *Slackia* | 0.00008 | 0.00008 | 0.00007 | . | . | . | . | . | . | . | . |
| *Sellimonas* | 0.00008 | 0.00003 | 0.00019 | . | . | . | . | . | . | . | . |
| *Allisonella* | 0.00008 | 0.00002 | 0.00012 | . | . | 0.04198 | . | . | . | . | . |
| *Mucispirillum* | 0.00007 | 0 | 0 | 6.11E-09 | 1.01E-07 | 5.49E-06 | 0.00009 | . | . | 8.84E-06 | 0.00013 |
| *Anaerotruncus* | 0.00007 | 0.00001 | 0.00005 | 0.00261 | 0.01314 | 0.00058 | 0.00538 | 0.02334 | . | . | . |
| *Rikenellaceae_RC9_gut_group* | 0.00006 | 0 | 0 | 1.51E-09 | 2.79E-08 | 2.33E-06 | 0.00005 | . | . | 3.71E-06 | 0.00006 |
| *Holdemania* | 0.00006 | 0.00002 | 0.00004 | . | . | . | . | . | . | . | . |
| *Oscillospira* | 0.00005 | 0.00004 | 0.00002 | . | . | . | . | . | . | . | . |
| *Peptococcus* | 0.00005 | 0.00006 | 0.00002 | . | . | . | . | . | . | . | . |
| *Macellibacteroides* | 0.00005 | 0 | 0 | 2.47E-08 | 3.41E-07 | 0.00001 | 0.00020 | . | . | 0.00002 | 0.00024 |
| *Eggerthellaceae_unclassified* | 0.00005 | 0.00003 | 0.00004 | . | . | . | . | . | . | . | . |
| *Paludicola* | 0.00004 | 0.00002 | 0.00002 | . | . | . | . | . | . | . | . |
| *UC5_1_2E3* | 0.00004 | 6.45E-06 | 0.00004 | . | . | . | . | . | . | . | . |
| *Lactococcus* | 0.00003 | 0.00002 | 0.00021 | . | . | . | . | 0.02510 | . | . | . |
| *Bacteria_unclassified* | 0.00003 | 0.00002 | 9.68E-06 | 0.01611 | . | . |  | . | . | 0.00338 | 0.02339 |
| *Candidatus_Stoquefichus* | 0.00003 | 0.00012 | 0.00006 | . | . | . | . | . | . | . | . |
| *Eubacterium* | 0.00003 | 0.00002 | 0.00003 | . | . | . | . | . | . | . | . |
| *Mogibacterium* | 0.00003 | 0.00008 | 4.30E-06 | . | . | . | . | . | . | . | . |
| *Succinatimonas* | 0.00003 | 0.00005 | 0.00005 | . | . | . | . | . | . | . | . |
| *Acetatifactor* | 0.00003 | 0 | 0 | 0.00002 | 0.00014 | 0.00065 | 0.00538 | . | . | 0.00052 | 0.00432 |
| *DTU089* | 0.00003 | 2.15E-06 | 6.45E-06 | 0.01138 | 0.04344 | 0.01110 | . | . | . | 0.03444 | . |
| *Angelakisella* | 0.00003 | 3.23E-06 | 0.00001 | . | . | . | . | . | . | . | . |
| *Tannerellaceae_unclassified* | 0.00003 | 0.00004 | 0.00001 | . | . | . | . | . | . | . | . |
| *UCG_009* | 0.00003 | 0.00002 | 0.00005 | . | . | . | . | . | . | . | . |
| *UCG_008* | 0.00002 | 2.15E-06 | 0.00003 | 0.01178 | 0.04346 | 0.00635 | 0.03720 | . | . | . | . |
| *Lactonifactor* | 0.00002 | 4.30E-06 | 0.00004 | . | . | . | . | . | . | . | . |
| *Defluviitaleaceae_UCG_011* | 0.00002 | 0.00002 | 0.00003 | . | . | . | . | . | . | . | . |
| *Oxalobacter* | 0.00002 | 0 | 1.08E-06 | 0.00780 | 0.03321 | 0.01069 | . | . | . | 0.03584 | . |
| *Anaerofilum* | 0.00002 | 0.00002 | 0.00003 | . | . | . | . | . | . | . | . |
| *Caproiciproducens* | 0.00002 | 5.38E-06 | 0.00002 | . | . | . | . | . | . | . | . |
| *Coprobacillus* | 0.00002 | 3.23E-06 | 6.45E-06 | . | . | . | . | . | . | . | . |
| *Weissella* | 0.00002 | 0.00005 | 0.00014 | . | . | . | . | . | . | . | . |
| *Candidatus_Soleaferrea* | 0.00001 | 8.60E-06 | 0.00003 | . | . | . | . | 0.02807 | . | . | . |
| *Gordonibacter* | 0.00001 | 0.00005 | 0.00003 | . | . | . | . | . | . | . | . |
| *Raoultibacter* | 0.00001 | 0.00002 | 0.00002 | . | . | . | . | . | . | . | . |
| *Frisingicoccus* | 0.00001 | 6.45E-06 | 0.00002 | . | . | . | . | . | . | . | . |
| *Christensenella* | 0.00001 | 2.15E-06 | 0.00002 | . | . | . | . | 0.05495 | . | . | . |
| *Firmicutes_unclassified* | 0.00001 | 5.38E-06 | 0.00002 | . | . | . | . | . | . | . | . |
| *Lachnospiraceae_UCG_003* | 0.00001 | 0.00005 | 0.00001 | . | . | . | . | . | . | . | . |
| *Phocea* | 0.00001 | 3.23E-06 | 0.00002 | . | . | . | . | . | . | . | . |
| *Mailhella* | 0.00001 | 3.23E-06 | 0.00003 | . | . | . | . | . | . | . | . |
| *Rothia* | 0.00001 | 0.00001 | 0.00003 | . | . | . | . | . | . | . | . |
| *Bifidobacteriaceae_unclassified* | 0.00001 | 9.68E-06 | 0.00001 | . | . | . | . | . | . | . | . |
| *Anaerostignum* | 4.30E-06 | 9.68E-06 | 8.60E-06 | . | . | . | . | . | . | . | . |
| *Granulicatella* | 3.23E-06 | 0 | 0.00003 | . | . | . | . | . | . | . | . |
| *Marivita* | 0 | 0 | 0.00006 | 2.47E-08 | 3.41E-07 | . | . | 0.00001 | 0.00030 | 0.00002 | 0.00021 |
| *Microbacteriaceae_unclassified* | 0 | 0 | 0.00016 | 1.84E-16 | 1.53E-14 | . | . | 7.44E-11 | 5.91E-09 | 1.27E-10 | 1.05E-08 |
| *Rhodobacteraceae_unclassified* | 0 | 0 | 0.00246 | 1.18E-19 | 1.96E-17 | . | . | 4.76E-13 | 7.58E-11 | 8.89E-13 | 1.48E-10 |

Supplementary Table S2. Means, medians, standard deviations, standard errors of standardized clinical scores at each time point (N=46). Wilcoxon signed-rank test was used to compare clinical scores between timepoints and to calculate p-values.

|  |  | **Standarized scores** | | | | **p-values** | | |
| --- | --- | --- | --- | --- | --- | --- | --- | --- |
|  | **Time** | **Mean** | **Median** | **Std. Deviation** | **Std. Error** | **D0 vs. D7** | **D0 vs. D21** | **D7 vs. D21** |
| **Frequency** | **D0** | 0.67 | 1.00 | 0.52 | 0.08 | 0.358 | 0.039 | 0.345 |
|  | **D7** | 0.76 | 1.00 | 0.48 | 0.07 |  |  |  |
|  | **D21** | 0.83 | 1.00 | 0.38 | 0.06 |  |  |  |
| **Gas** | **D0** | -1.15 | 0.00 | 0.63 | 0.09 | 0.750 | 0.043 | 0.005 |
|  | **D7** | -0.17 | 0.00 | 0.74 | 0.11 |  |  |  |
|  | **D21** | 0.13 | 0.00 | 0.86 | 0.13 |  |  |  |
| **Energy** | **D0** | 0.59 | 1.00 | 0.91 | 0.13 | 0.011 | 0.006 | 0.227 |
|  | **D7** | 0.91 | 1.00 | 0.84 | 0.12 |  |  |  |
|  | **D21** | 1.02 | 1.00 | 0.83 | 0.12 |  |  |  |
| **Bristol** | **D0** | -0.59 | 0.00 | 0.69 | 0.10 | 0.292 | 0.078 | 0.745 |
|  | **D7** | -0.46 | 0.00 | 0.62 | 0.09 |  |  |  |
|  | **D21** | -0.41 | 0.00 | 0.62 | 0.09 |  |  |  |

**Supplementary Figures**


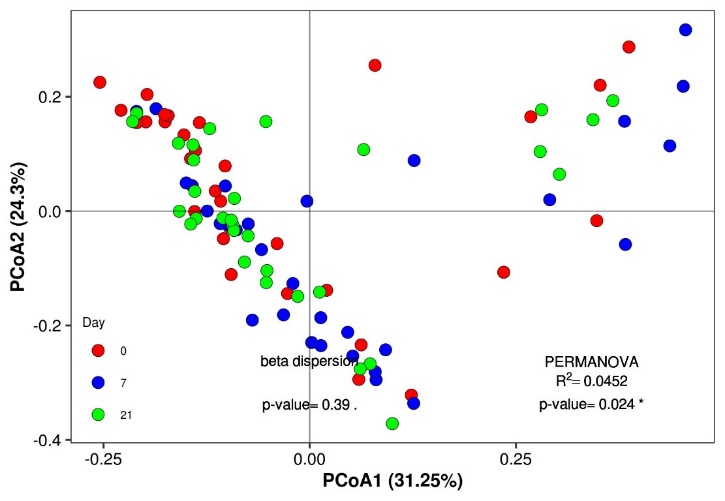


Supplementary Figure S1. PCoA plot of beta diversity of the gut microbiota based on weighted Bray-Curtis distance matrix. Samples are color coded by timepoint.


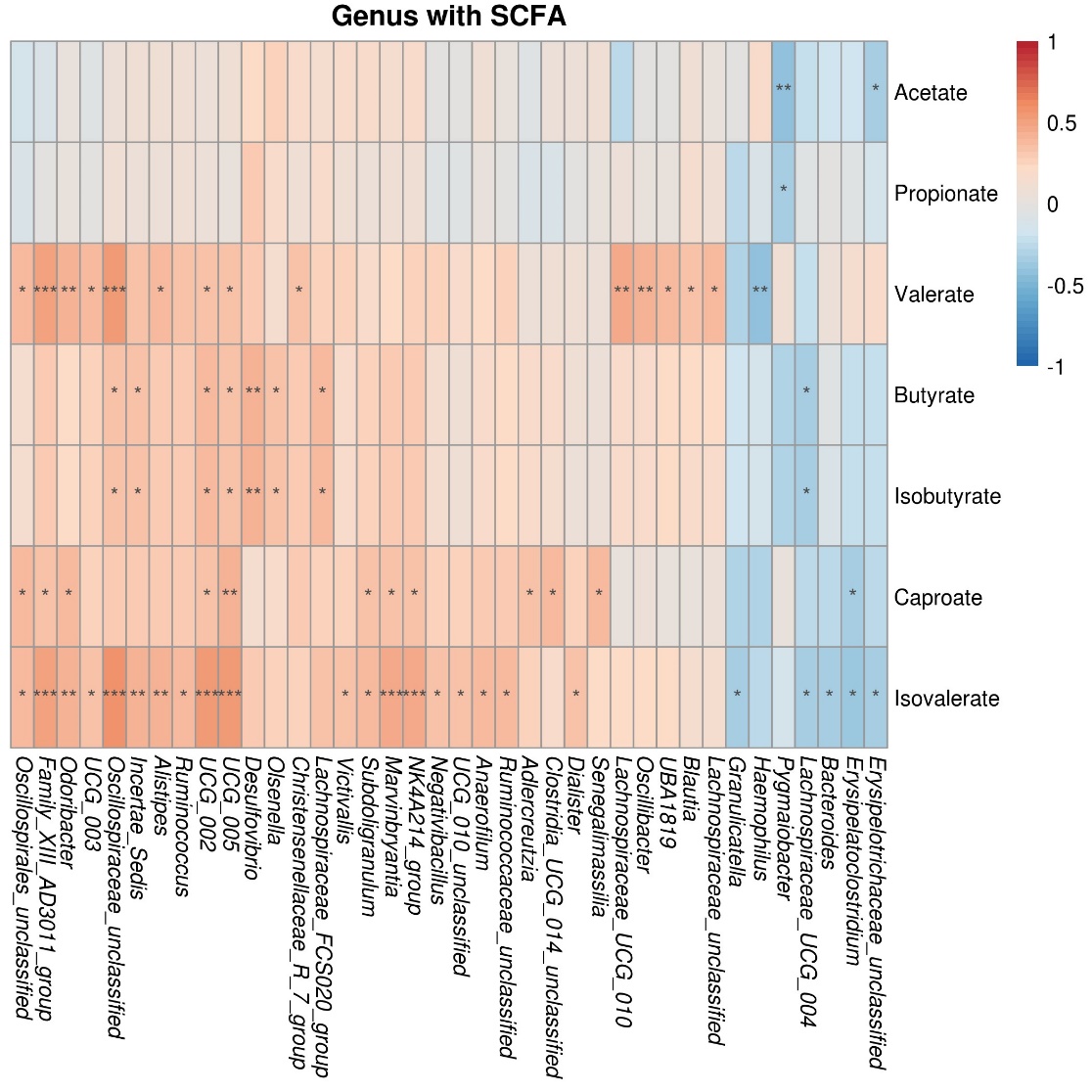


Supplementary Figure S2. Heatmap of Spearman correlations between genus level taxa and each SCFA concentration. Displayed are taxa for which at least one correlation passed a threshold of >±0.3 and statistical significance calculated using Wilcoxon signed-rank test, with FDR p-values corresponded to *≤ 0.05, ** ≤ 0.01, *** ≤ 0.001.
